# Economic inequality increases the number of hours worked and decreases work–life balance perceptions: longitudinal and experimental evidence

**DOI:** 10.1098/rsos.230187

**Published:** 2023-10-18

**Authors:** Silvia Filippi, Bruno Gabriel Salvador Casara, Davide Pirrone, Mara Yerkes, Caterina Suitner

**Affiliations:** ^1^ Department of Developmental Psychology and Socialization, University of Padova, Padova, Italy; ^2^ Science Division, NYU Abu Dhabi, Abu Dhabi, UAE; ^3^ Department of Experimental Clinical and Health Psychology, University of Ghent, Ghent, Belgium; ^4^ Department of Interdisciplinary Social Sciences, Utrecht University, Utrecht, Netherlands

**Keywords:** work–life balance, economic inequality, socio-economic class, status anxiety, competitiveness

## Abstract

International institutions' attention to work–life balance (WLB) demonstrates the global breadth of this issue. Yet the scientific community has thus far paid little attention to its structural underpinnings and to the interplay between these macro-level underpinnings and individual psychological factors. We examine the contextual role of economic inequality at the national level as a significant factor influencing working time and WLB perceptions using multiple empirical strategies. In the first set of studies (1a and 1b), we compared countries with different levels of inequality (Study 1a with 37 countries, Study 1b with longitudinal data from 34 countries, *N* = 254) and found increased working time and reduced WLB in highly unequal countries. In a pilot study (*N* = 81) and in the pre-registered Studies 2 (*N* = 338) and 3 (*N* = 499) we corroborated this evidence with an experimentally induced inequality perception, reporting an indirect effect of inequality on WLB (Studies 2 and 3) and working time (Study 3) through status anxiety and competitiveness. In Study 2, we manipulated socio-economic class in addition to economic inequality, showing that the detrimental effect of inequality on WLB is especially marked for participants assigned to a low-class condition. This research contributes to an integrated understanding of the impact of economic inequality and socio-economic class in shaping WLB and provides useful insights for organizations to develop context-specific policies to improve employees’ WLB that take both individual and structural factors into account.

## 1. Economic inequality increases the number of hours worked and decreases work–life balance perceptions: longitudinal and experimental evidence

Achieving a satisfactory balance between work and personal life [1] is a policy priority within several international institutions, including the European Union, the World Health Organization, and the International Labour Organization. Despite this attention, the extant literature and subsequently available policies focus primarily on organizational causes and individual-level consequences of work–life interference, with insufficient attention for the structural, socio-economic foundations of work–life balance (WLB) [2–8]. Drawing on Bronfenbrenner's social-ecological model [9], we suggest macro variables are needed to understand organizational phenomena [10], viewing organizational issues in relation to national features. Although a wealth of literature examines national-level work–life policies (e.g. [11,12]), only few studies explicitly address macro-level drivers of WLB ([13] for an exception on the importance of gender inequality for WLB).

Here we argue that an important factor characterizing socio-economic context and affecting WLB is economic inequality (henceforth inequality), defined as ‘the unequal dispersion of resources across society’ [14]. Inequality is increasing in most Organisation for Economic Co-operation and Development (OECD) countries [15] and is expected to increase even further given rising food and energy prices and a decline in wages, causing growing inequality between households [16]. Such inequality is linked to increased burnout and job insecurity [17] and to reduced well-being in general [18,19]. Despite constant increase in inequality, research on the implications of inequality for organizational contexts remains limited (see [17] for an exception), although strongly suggested by inequality scholars (see for example [20]).

From a psychological point of view, working is inextricably linked to the demand for economic means to meet basic needs and enhance social status [21]. Literature shows that, in a highly unequal context, people perceive greater instability related to their job situation [17], increased competition with other citizens and enhanced concerns related to their position on the social ladder (status anxiety; [22]). These feelings are potentially important in shaping how individuals experience the allocation of time between work activities and their personal life.

We provide an empirical contribution by offering an integrative quantitative analysis of the relationship between inequality (both at the actual and perceived levels), working hours and WLB. The need to analyse both actual inequality at the macro level and individual perceptions of inequality arising from literature, demonstrating that the effects of actual inequality are in general weaker compared with the ones of perceived inequality [23–25], although related [26]. Moreover, we study psychological processes that may explain this link, namely perceived status anxiety and competitiveness. From a practical standpoint, these findings may help organizations develop context-specific and more accurate WLB policies that take both individual and contextual factors into account.

## 2. The role of inequality, status anxiety and competitiveness in shaping working time and work–life balance perceptions

WLB is a contested concept (e.g. [27,28]). It implies that work can be brought to an equilibrium with activities outside of work [29], yet meanings of ‘balance’ differ, with scholars alternatively using concepts of work–life fit, satisfaction or effectiveness [30]. While many authors focus on the work component of this equilibrium (e.g. [28]), others stress the life part, emphasizing valued time and effort spent on multiple life activities [31]. Similar to its conceptualizations, the way WLB is empirically handled in the literature varies widely [32,33]. Measures of WLB are usually used to capture individual experiences of work in relation to family and private life, such as measures of work–life conflict [34], work–life fit [35], or work/non-work interference (e.g. [36]). Hours worked is sometimes used as an indicator of WLB [1]. The extent to which material reality (hours worked) is related to WLB is complex, however [32]. Some evidence suggests that long working hours jeopardize work–life balance [37], an effect that was observed across 17 studies [38]. However, in some reports, the association is the opposite, with workers that are satisfied with their job and their life being more likely to work longer hours [39]. It, therefore, remains an empirical question whether working hours could be used as a reliable proxy of WLB and whether it is similarly affected by contextual factors such as economic inequality.

Despite limited attention to the relationship between inequality and WLB, indirect evidence points to a plausible relationship and the need for further study. For example, Wilkinson and Pickett [40] posited that inequality creates a specific environment in which people must adapt their behaviour strategically. Sánchez-Rodríguez *et al*. [41] take this idea one step further by suggesting that inequality influences individual perceptions of a society's normative climate. Inferring those norms may therefore guide attitudes, emotions and behaviours (see the economic inequality as normative information model (EINIM)). Which normative climate is perceived in a context of high inequality, and which behaviours are triggered under such circumstances? Extant literature shows that inequality stresses the differentiation of individuals into social classes [42,43] and promotes social comparison [44]. With categorizations based on wealth becoming more salient [45], people are more likely to feel deprived [46] and less wealthy [47] compared with others. Inequality breeds the perception of a competitive normative climate [41,48] in which people's behaviour is driven by concern over their position on the social ladder (status anxiety; [22,49]).

One correlational study by Bowles and Park [50] has provided initial evidence of the link among inequality, status anxiety and the number of hours worked. This study shows that in countries with higher levels of inequality, people tend to work more, as a result of a desire for social standing, indirectly measured through consumption habits. The authors specifically suggested that people's consumption habits and work choices reflected a desire to feel valued and to distinguish themselves from a disadvantaged reference group. Despite this, evaluating consumption patterns as a direct measure of status anxiety may not be totally accurate, as some data suggests that it is rather a consequence of it (e.g. [51]). The role of status anxiety in this relationship, thus, needs to be further explored. Complementary evidence links inequality to status anxiety via competitiveness [22], but a clear understanding of the pathways connecting inequality to WLB remains unknown. To fill this gap, we here argue that both status anxiety [52] and competitiveness would lead people to work longer hours and perceive a decreased balance between work and private life. Moreover, we explore the role of socio-economic class in this relationship.

## 3. The role of socio-economic class in shaping status anxiety and work–life balance

According to the literature, both upper- and lower-class belonging may be a risk factor for status anxiety and its correlated WLB.

More specifically, upper-class individuals often face demanding work environments characterized by long working hours, intense job responsibilities and accessibility [53], and high career expectations [54]. This pressure to high achievements that characterizes individuals from wealthy classes since their childhood may turn into high anxiety levels [55]. Moreover, the prestigious positions and financial rewards of wealthy workers may jeopardize their ability to separate work-related obligations from personal life [56], motivated by the desire to maintain their high-status positions and by the practical flexibility they have on their schedule allowing for overworking [57]. Concerning the lower class, there are obvious reasons for low-income people to cover their material needs with increased working hours because of economic precarity (e.g. [21,58,59]). Along the same line, a worsened WLB can be explained by objective burdens, such as lower control over their working schedule [60], limited access to benefits such as paid leave and flexible work arrangements [21] or inability to outsource childcare, housework or other tasks [61]. However, there are also social-psychological drives. Specifically, status anxiety may be central for explaining a tendency to overwork, at the expense of other parts of life, as low-income people may face higher general anxiety levels stemming from the economic burdens they need to face in their daily life. More specifically, low-status people experience high levels of status anxiety as they not only are by definition lacking social status, but they also feel devaluated because of their socio-economic position [62]. The link between status anxiety and reduced WLB among low-income people is not only explained by the need to secure sufficient income and provide for basic necessities, which may require low-income individuals to work multiple jobs or extended hours, leaving limited time for personal life [63], but also the desire of improving the social position.

Is sum, high- and low-income people work longer hours and sacrifice their work–life balance motivated by opposite and complementary reasons stemming from status anxiety, the first being motivated by the need to maintain their privileged economic and status position, the second by material needs and the hope to obtain more social status and thus avoid to feeling devaluated.

In a context of high inequality, the aforementioned pattern should be polarized because of the enhanced segregation, psychological distinction and, therefore, salience of the socio-economic classes [42], as the low-income group is less wealthy and the high-income group is wealthier. Under high inequality, upper-class individuals may feel more pressure, possibly because they face greater social pressures to maintain higher incomes and generally have greater professional responsibilities [64].

Inequality also intensifies the financial hardships of low-status individuals [65] and increases concerns about one's position in the social ladder—in other words, the feeling of being (versus not being) valued in the eyes of others [44,62,66]; —and competitiveness among individuals [22].

## 4. The present work

The primary goal of the present paper was to directly test the theorized negative relationship between inequality and WLB. To unpack the dynamics between these two variables, we considered both micro (individual socio-economic status and individual perceptions of inequality at the national level) and macro (actual level of inequality) mechanisms, in line with Hobson and colleagues' view that individual perceptions of WLB are situated in varying national contexts [31]. To that end, we undertook four studies entailing different samples, multiple empirical strategies (cross-sectional, longitudinal and experimental), and different indicators of WLB (including behavioural proxies such as work hours and self-reported scales of work/non-work interference). We did this accounting for both objective and perceived inequality. In the first two studies (Studies 1a and 1b), we investigated the macro-level association between objective inequality and WLB, both cross-sectionally and longitudinally, using annual working hours to account for the absence of cross-country longitudinal data on WLB. In a pilot study, we corroborated this link by experimentally testing the role of perceived inequality in decreasing WLB through the mediation of status anxiety.

Furthermore, to extend the limited evidence on this topic, we aimed to understand whether living in a context perceived to be economically unequal poses different challenges to people belonging to different socio-economic classes. To explore the effect of socio-economic class in this relationship, we ran the pre-registered experimental Study 2, where we aimed to replicate the effect of inequality on WLB (H1) and further explored the role of socio-economic class. We hypothesized that In Study 3, we replicated the path linking inequality, working hours and WLB using a high-powered sample and pre-registered hypotheses. Data files and materials associated with the manuscript are openly available online on OSF (https://osf.io/3a6w9/?view_only=eb5e75fb0cff4314b31574588aca7f9f).

## 5. Studies 1a and 1b

The goal of Studies 1a and 1b was to explore the relationship between objective inequality and WLB using large-scale datasets. We hypothesized that inequality would be negatively correlated with WLB (Study 1a) and positively with work hours (Study 1b).

### 5.1. Methods

For Study 1a, WLB information of 37 countries was gathered from the Better Life Index [67], which is based on public use of time survey microdata (e.g. Eurostat's Harmonised European Time Use Surveys), and tabulations from National Statistical Offices and the OECD's labour force statistics. The WLB indicator is calculated by the OECD using two observable variables: (i) the country average for the time devoted to leisure and personal care activities, and (ii) the percentage of dependent employees who work more than 50 h per week. Data for employees working long hours are then transformed to get an indicator of ‘no long hours worked’, and then computed together with the time devoted to leisure and personal care, to construct the WLB indicator (ranging from 0 = low WLB to 10 = high WLB).

In Study 1b, we analysed the longitudinal relationship between inequality and the number of hours worked. Data measuring individual-level average annual working hours (per week) were retrieved from the website Our World in Data. The dataset contained data from 1981 to 2017 for 34 countries.

Data related to the Gini index and the GDP per capita were gathered from the World Bank estimates and matched per year and country (2017; from 2014 for Study 1a; from 1967 to 2014 for Study 1b). The Gini index ranges on a scale from 0 (perfect equality, everyone has the same level of wealth) to 100 (perfect income inequality, just one person owns all the wealth in a country). This dataset contained 277 Gini scores ranging from 1967 to 2014.

For Study 1b, we selected data from countries that had at least four annual Gini observations available. Given missing data, the final sample was *N* = 265 observations from 34 countries.

Finally, in both studies, we tested the relationship between Gini and working hours controlling for GDP per capita.

### 5.2. Results

#### 5.2.1. The effect of economic inequality on work–life balance

**Figure 1.**
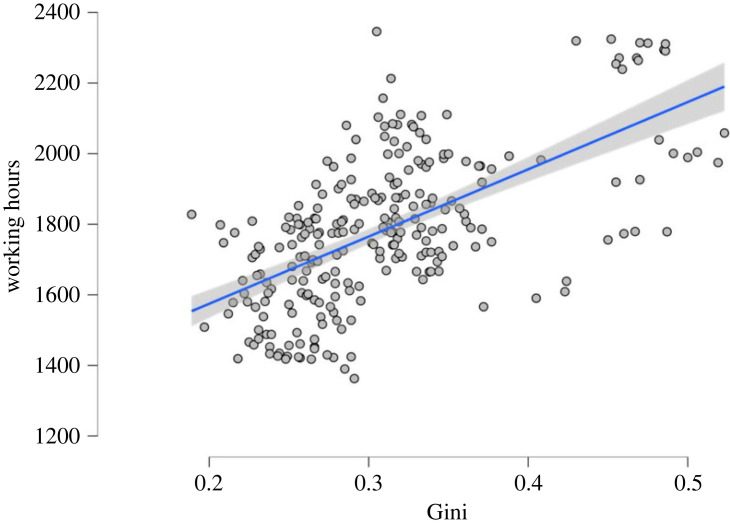
Effect plots on the effect of Gini index, on annual working hours, controlled for year, GDP per capita and country from Study 1b. Grey areas represent 95% confidence intervals.

Analyses were conducted using the software R (v. 4.1.3). In Study 1a, to test the effect of inequality on WLB, we ran a multiple linear regression. The Gini coefficient negatively correlates with WLB (*b* = –0.13; s.e. = 0.04, *p* < 0.001), meaning higher inequality is associated with lower levels of WLB, controlling for country-specific GDP per capita. Similar results were found in Study 1b (figure 1). Here, a mixed-effects model was used to test our hypothesis. This model allows us to test the association between country-level Gini coefficients and individual annual average working hours by controlling for both the different years of observation, GDP per capita, and country differences (allowing for random intercepts). Results suggest that while individual average annual working hours decreased across time (*b* = 5.06; s.e. = 0.53, *p* < 0.001), a higher annual average of working hours is associated with higher Gini coefficients and thus higher inequality (*b* = 4.39; s.e. = 0.80, *p* < 0.001). In this study, GDP per capita was not a significant predictor of the number of average annual working hours (*b* < 0.001; s.e. < 0.001, *p* = 0.10).

### 5.3. Discussion

Study 1a shed initial light on the detrimental effect of inequality on WLB. This replicates the effect found in the correlational study by Bowles and Park [50], investigating 10 countries, and extends it to a larger sample of 37 countries, including non-Western countries. Moreover, it further corroborates the pattern with a more accurate measure of WLB, provided by the OECD Better Life Index [67]. Study 1b further improves the quality of the available data as it involves longitudinal observations for 34 countries across 36 years, allowing for 254 data points. Results of Study 1b revealed the same pattern as Study 1a, with inequality increasing the average number of hours worked per year. Studies 1a and b have strong ecological validity as they capture real data on a large scale. Yet no causal inferences can be drawn as Study 1a is cross-sectional and Study 1b was not carried out in a controlled setting. Indeed, we recognize that while we have considered multiple relationships among structural, macro-level variables, potential mediating variables may be explored in future research. For example, the unemployment rate (e.g. [68,69]) or union density (e.g. [70]) are elements that are associated with inequality and have an obvious relevance for the number of worked hours. In a pilot study and in two well-powered pre-registered studies, we tested the impact of inequality on WLB using an experimental methodology that can directly test the causal relationship between inequality and expected WLB.

## 6. Study 2

In a first pilot study (*N* = 81), we experimentally tested the link between macro-level inequality and individual-level expected WLB initially evidenced by Studies 1a and 1b, and explored a possible explanation of this effect using status anxiety as a mediator (see pilot study in the electronic supplementary material). The results of the pilot study provided initial experimental evidence on the path linking inequality with decreased WLB, supporting cross-sectional and longitudinal data presented in Studies 1a and 1b. Study 2 was built to replicate results found in the pilot study with a larger sample size. Moreover, we explored the effect of competitiveness as another potential mediating variable and the role of socio-economic class in the relationship between inequality and WLB [71]. Study 2 was pre-registered on the platform AsPredicted.com (https://aspredicted.org/blind.php?x=jp9uk9). As in the pilot study, we used the Bimboola paradigm [72] to manipulate the perception of inequality. Moreover, we randomly assigned each participant to a socio-economic class (lower, middle or upper). We pre-registered five main hypotheses. Following Study 2, we predicted that participants assigned to the high (versus low) inequality condition will expect lower WLB (H1), and more status anxiety (H2). Moreover, we predicted that participants assigned to lower and upper classes would perceive lower expected WLB and higher expected status anxiety (H4), compared with participants assigned to the middle class, in line with past, though mixed, literature [73,74]. In addition, we predicted that expected WLB would be negatively associated with expected status anxiety (H4). Furthermore, we explored the effect of competitiveness and the indirect effects of inequality via status anxiety and competitiveness on expected WLB as exploratory hypotheses. As an exploratory analysis, we also tested the potential interaction effect between inequality and socio-economic class.

### 6.1. Method

#### 6.1.1. Participants

Italian participants were collected using a snowball sampling procedure though a Qualtrics link disseminated online by social psychology students, with the goal of having at least 240 participants in line with our pre-registered desired sample size. The stopping rule coincides with the time frame allocated to data collection (from 26 November to 6 December 2020) for teaching reasons. Participation in the study was fully voluntary and participants did not receive any compensation. Following data cleaning (using the same exclusion criteria as in the pilot study), we obtained a final sample size of 338 (68.6% women; *M*_age_ = 31.8; s.d. = 14.2, age ranging from 18 to 79; more information about the sample is presented in table 1).

**Table 1.** Demographic characteristics of the three experimental samples.

	sample size	gender	age	political orientation	employment status	socio-economic status	education
pilot study	81	F = 87.7%	M = 21.06	M = 3.41	student (87.7%)	lower class (2.5%)	high school diploma (90.1%)
		M = 12.3%	s.d. = 2.92	s.d. = 2.10	working student (9.9%)	lower-middle class (22.2%) Middle class (42.0%)	bachelor's degree (3.7%)
		NB = 0%		(0 = extreme left, 10 = extreme right)	employee (1.2%)	upper-middle class (33.3%)	master's degree (3.7%)
						upper class (0%)	PhD (2.5%)
Study 2	338	F = 68.6%	M = 31.80	M = 4.80	employee (45.5%)	lower class (1.5%)	primary/middle school (3.3%)
		M = 31.4	s.d. = 14.2	s.d. = 2.30	self-employed (10.9%)	lower-middle class (23.1%) Middle class (55.3%)	high school diploma (49.4%)
		NB = 0%		(0 = extreme left, 10 = extreme right)	student (40.5%%)	upper-middle class (19.8%)	bachelor's degree (15.1%)
						upper class (0.3%).	master's degree (26.6%)
							PhD (5.6%)
Study 3	499	F = 48.3%	M = 31. 34	M = 3.33	employee (38.5%)	lower class (3.6%)	mandatory school (1.6%)
		M = 49.7%	s.d. = 10.03	s.d. = 1.98	self-employed (14.4%)	lower-middle class (29.3%)	middle school (0.4%)
		NB = 2%		(0 = extreme left, 10 = extreme right)	student (32.7%%)	middle class (55.9%)	high school diploma (41.7%)
					unemployed (12.6%)	upper-middle class (11.2%)	bachelor's degree (27.5%) master's degree (23.4%)
					other (1.8%)	upper class (0%)	PhD (5.4%)

To calculate sample size, we performed an *a priori* power analysis partially based on the effects found in the pilot study, with *β* = 0.80 and *α* = 0.05, using the package paramtest [75], simulating a multiple linear regression with two main effects: inequality manipulation and socio-economic class manipulation. We fixed the main effect of the inequality manipulation at b1 = 0.5, the main effect of the high socio-economic class manipulation at b2 = 0.3, and the main effect of the low socio-economic class manipulation at b3 = 0.6. The results of a simulation with 5000 iterations showed that *N* = 240 was required to achieve the desired power for these effects, which was our pre-registered sample size goal.

#### 6.1.2. Materials and procedure

The survey was hosted on the Qualtrics platform. Unless otherwise specified, the response options of measures ranged from 0 = strongly disagree to 10 = strongly agree on a 10-point scale.

##### 6.1.2.1. Perceived economic inequality

After signing informed consent, participants were asked to imagine that they were going to start a new life in a fictitious society named Bimboola, whose income distribution varied in two conditions to which participants were randomly assigned:

— In the high-inequality condition, Bimboola was characterized by three income groups, which differed greatly in the average annual income earned expressed in Bimboolean dollars per year (lower class = 3000; middle class = 40 000; upper class = 70 000).
— In the low-inequality condition, the three income groups differed slightly in their annual earnings expressed in Bimboolean dollars per year (lower class = 30 000; middle class = 40 000; upper class = 50 000).

The procedure was the same as the pilot study, but in addition to the inequality manipulation (high versus low), we also manipulated socio-economic status by randomly placing participants in three different groups: upper, middle, and lower class (as in [76]). This results in a 2 (high versus low inequality) × 3 (lower versus middle versus upper class) between-participant design. For further details on the manipulation, see materials provided on OSF. Participants then answered four manipulation-check questions regarding the economic standing of the group they were assigned to (‘my group is poor’ and ‘my group is rich’, *r* = −0.93) and income inequality in Bimboola (‘Income differences between Bimboola's citizens are low’ and ‘Income differences between Bimboola's citizens are high’, *r* = −0.77).

##### 6.1.2.2. Expected work–life balance

Expected WLB was assessed with the subscales WIPL and PLIW of the Work/Non-work Interference and Enhancement Scale [77], translated into Italian (*α* = 0.91). The subscale WIPL assesses work interference with personal life (e.g. ‘I come home from work too tired to do things I would like to do’), while the subscale PLIW assesses personal life interference with work (e.g. ‘When I am at work, I worry about things I need to do outside work’). We calculated the mean of the two subscales and reversed it to obtain a measure of work–life balance.

##### 6.1.2.3. Expected status anxiety

We used an Italian adaptation of Dehley *et al.*'s [44] status anxiety scale (e.g. ‘Some people look down on me because of my job situation or income’, and ‘I do not feel that the value of what I do is recognized by others’, *r* = 0.70).

##### 6.1.2.4. Expected competitiveness

We also assessed expected competitiveness with an Italian adaptation of the 5-item scale from Murayama and Elliot [78], ‘In Bimboola, it seems that people are competing with each other’; ‘People seem to share the feeling that competing with each other is important’; *α* = 0.86).^1^

As a difference from the pilot study, participants were asked to report their own expected WLB (subscales WIPL and PLIW^2^), status anxiety and competitiveness as a Bimboolean citizen, rather than their attributions about a general Bimboolean citizen. Social support and need for achievement were included in the pre-registration as control variables, without any specific hypotheses. Given that the pattern of the results is similar if we include or not these variables as covariates, for parsimony they are not further discussed in the manuscript.

### 6.2. Results

#### 6.2.1. Manipulation checks

Perceived wealth was mostly affected by class manipulation, *F*_2, 332_ = 641.31; *p* < 0.001, $\eta_{p}^{2}=0.794$. However, also inequality had a small effect on perceived wealth, *F*_1, 332_ = 10.17; *p* = 0.002, $\eta_{p}^{2}=0.03$.

Perceived inequality was affected by inequality condition *F*_1, 332_ = 224.491; *p* < 0.001, $\eta_{p}^{2}=0.40$ but not by class manipulation, *F*_2, 332_ = 2.32; *p* = 0.10, $\eta_{p}^{2}=0.004$ (complete descriptives are presented in electronic supplementary material, tables S4 and S5).

#### 6.2.2. Main analyses

We ran three separate ANOVAs [79] including expected WLB, expected status anxiety or expected competitiveness as dependent variables, and inequality and socio-economic class as predictors. In all *post hoc* analyses, *p*-values are adjusted using Tukey's correction.

#### 6.2.3. Effect of inequality and socio-economic class on expected work–life balance

**Figure 2.**
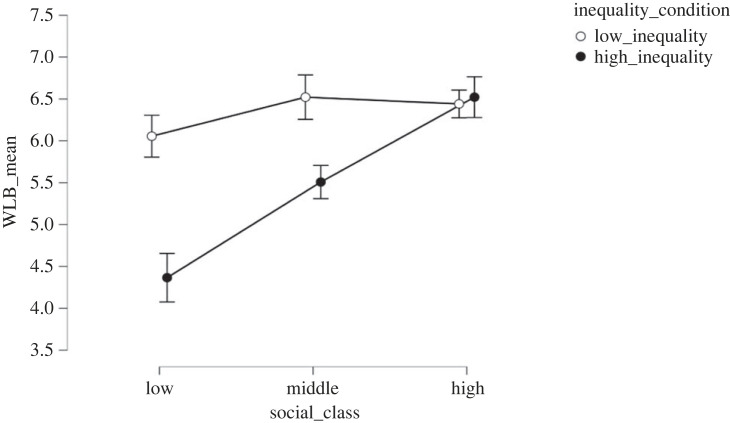
Effect of economic inequality and socio-economic class conditions on expected WLB in Study 2.

Expected WLB was affected both by inequality (*F*_1, 332_ = 20.58; *p* < 0.001, $\eta_{p}^{2}=0.05$) and class (*F*_2, 332_ = 14.13; *p* < 0.001, $\eta_{p}^{2}=0.02$) (figure 2). Expected WLB was lower in the high (*M* = 5.40; s.d. = 1.97) than low inequality condition (*M* = 6.35, s.d. = 1.68, *d* = 0.48), in line with H1 and the pilot study. Partially in line with H3, expected WLB was lower in the lower class (*M* = 5.19, s.d. = 2.13) compared with the middle (*M* = 5.93, s.d. = 1.85, *t* = 3.46, *d* = 0.41, *p* = 0.002) and upper (*M* = 6.47, s.d. = 1.44, *t* = 5.25, *d* = 0.70, *p* < 0.001) classes. There were no differences in expected WLB between middle and upper classes (*t* = 2.00, *d* = 0.28, *p* = 0.11).

Expected WLB was further characterized by the interaction between class and inequality (*F*_2, 332_ = 6.81, *p* = 0.001, $\eta_{p}^{2}=04$). Specifically, *post hoc* tests with Tukey correction highlighted that differences between inequality conditions were found for the middle (*M*_high inequality_ = 5.51; s.d._high inequality_ = 1.68; *M*_low inequality_ *=* 6.52; s.d._low inequality_ *=* 1.93; *t* = 3.21; *d* = 0.57; *p* = 0.02) and the lower classes (*M*_high inequality_ = 4.37; s.d*.*_high inequality_ = 2.12; *M*_low inequality_ *=* 6.06; s.d._low inequality_ *=* 1.36*; t* = 4.94; *d* = 0.86; *p* < 0.001), but not for the upper class (*M*_high inequality_ = 6.52; s.d._high inequality_ = 1.58; *M*_low inequality_ *=* 6.44; s.d._low inequality_ *=* 1.36; *t* < 0.3). Looking at the data from a different perspective, we can also see that in the high-inequality condition, participants assigned to the middle class reported lower levels of expected WLB compared with participants assigned to the upper class (*t* = −3.00; *d* = −0.62; *p* = 0.04), but higher levels compared with the lower class (*t* = 3.62; *d* = 0.61; *p* = 0.005). No differences between classes were found in the low inequality condition (all *d*_s_ *<* 0.25; all *p* > 0.05). To check the robustness of these results, we ran two ANCOVA models where we added age, income, level of education, gender, and subjective socio-economic status as covariates. The effects of the inequality condition (*d* = 0.48, *p* < 0.001) and the social class manipulation ($\eta_{p}^{2}=0.08$, *p* < 0.001) remained statistically significant.

#### 6.2.4. Effect of inequality and socio-economic class on expected status anxiety

**Figure 3.**
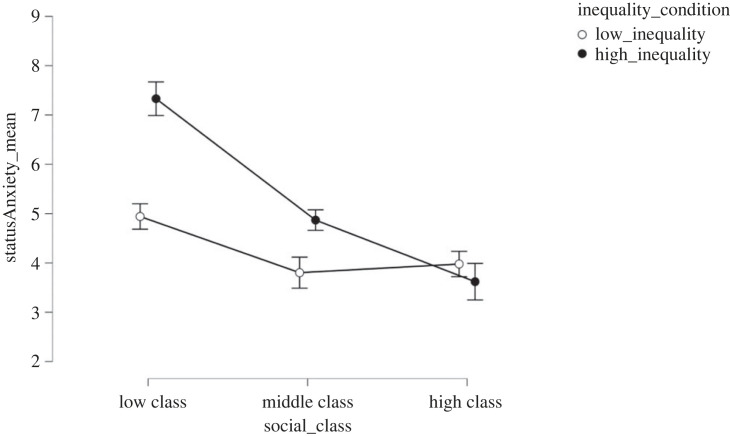
Effect of economic inequality and socio-economic class conditions on expected status anxiety in Study 2.

Both inequality (*F*_1, 332_ = 19.16; *p* < 0.001, $\eta_{p}^{2}=0.04$) and class (*F*_2, 332_ = 34.51; *p* < 0.001, $\eta_{p}^{2}=0.16$) significantly predicted status anxiety. In line with H2, expected status anxiety was smaller in the low (*M* = 4.21, s.d. = 2.13) compared with the high (*M* = 5.34, s.d. *=* 2.61) inequality condition (*t* = 4.54, *d* = 0.48, *p* < 0.001). H5 was partially supported as expected status anxiety was larger in the lower (*M* = 6.16, s.d. = 2.49) than the middle (*M* = 4.42, s.d. = 2.06, *t* = 6.33, *d* = 0.80, *p* < 0.001), and upper (*M* = 3.84, s.d. = 2.23, *t* = 7.89, *d* = 0.99, *p* < 0.001) classes. There were no differences in expected status anxiety scores between middle and upper classes (*t* = 1.88, *d* = 0.25, *p* = 0.15). Moreover, a significant interaction between class and inequality (*F*_2,332_ = 10.74; *p* < 0.001, $\eta_{p}^{2}=0.05$) also emerged (figure 3).

Differences between inequality conditions were found for the lower class (*t* = 5.71; *d* *=* 1.09; *p* < 0.001), but not for the middle (*t* = 2.76; *d* *=* 0.53; *p* = 0.07), or the upper classes (*t* = 0.85; *d* *=* 0.16; *p* = 0.96). However, no differences between classes were found in the low-inequality condition (*M*_low_ *=* 4.94; s.d. *=* 1.84; *M*_middle_ *=* 3.80; s.d. = 2.29; *M*_high_ *=* 3.98; s.d. = 2.11*,* all *p* *>* *0.05;* all *d* *<* 0.55). Furthermore, in the high-inequality condition, participants assigned to the middle class (*M* = 4.87; s.d. = 1.76) reported higher levels of expected status anxiety compared with participants assigned to the upper class (*M* = 3.62; s.d. = 2.41; *t* = 3.02; *d* = 0.62; *p* = 0.03), but lower levels of status anxiety compared with the lower class (*M* = 7.33; s.d. = 2.48; *t* = 6.38; *d* *=* −0.64; *p* < 0.001). The effects of inequality (*d* = 0.51, *p* < 0.001) and social class manipulations ($\eta_{p}^{2}=0.17$, *p* < 0.001) remained significant even after controlling for political orientation, income, subjective social class ('to which socio-economic class do you think you belong to?' 1 = lower class; 2 = lower-middle class; 3 = middle class; 4 = upper-middle class; 5 = upper class), gender and age. These findings suggest that the effect of inequality on expected WLB and expected status anxiety also depends on socio-economic class, affecting mainly the lower class, partially supporting H3.

#### 6.2.5. Effect of inequality and socio-economic class on expected competitiveness

**Figure 4.**
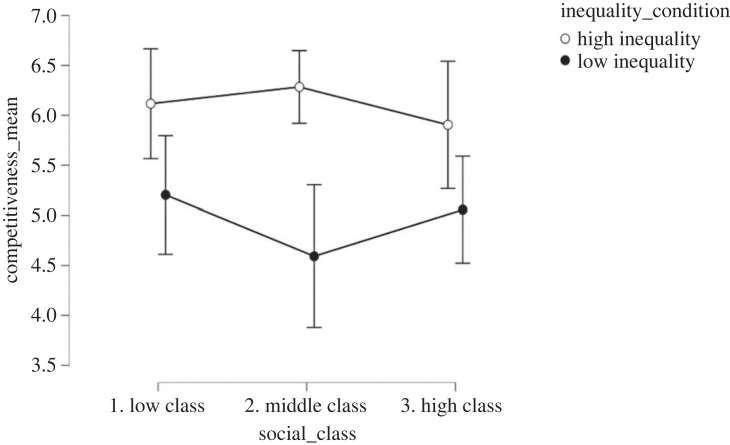
Effect of economic inequality and socio-economic class conditions on expected competitiveness in Study 2.

Inequality enhanced expected competitiveness (*M*_high inequality_ = 6.14, s.d._high inequality_ = 1.82, *M*_low inequality_ = 4.96, s.d._low inequality_ = 2.28, *d* = 0.52; *F*_1, 330_ = 25.13; *p* < 0.001, $\eta_{p}^{2}=0.07$). However, socio-economic class (*F*_2, 330_ = 0.350; *p* = 0.71, $\eta_{p}^{2}=0.002$) and the interaction between inequality and socio-economic class (*F*_2, 330_ = 1.46; *p* = 0.23, $\eta_{p}^{2}=0.008$) did not affect expected competitiveness scores (figure 4). The effect of inequality manipulation (*d* = 0.53, *p* < 0.001) remained significant even after controlling for political orientation, income, subjective social class, gender and age.

#### 6.2.6. Relationship between expected status anxiety and work–life balance

In line with H4, perceived status anxiety was negatively associated with WLB (*r* = −0.536; *p* = < 0.001).

#### 6.2.7. Mediation analysis

**Figure 5.**
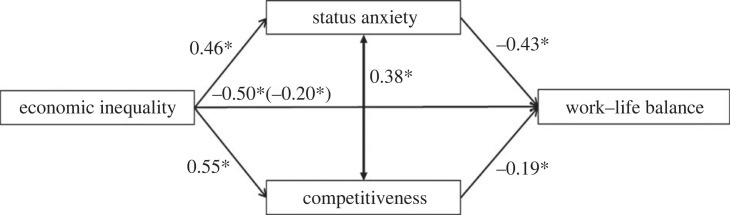
Mediation model examining indirect effects of economic inequality on expected WLB, through status anxiety and competitiveness in Study 2. Standardized coefficients are presented. Solid arrows and asterisks indicate significant paths (*p* < 0.05).

We examined whether expected status anxiety and competitiveness mediated the relationship between inequality and WLB using the software JASP [79] with bootstrapping for 5000 resamples and 95% confidence intervals [80]. *p*-values were corrected using the Benjamini–Hochberg correction [81]. In this model the inequality manipulation was the predictor variable, expected WLB the outcome variable, and expected status anxiety and competitiveness were the mediator variables (table 2). As shown in figure 5, we found two significant indirect effects of the inequality manipulation on expected WLB via expected status anxiety (*b* = −0.20, CI *=* [−0.31, −0.11]) and competitiveness (*b* = −0.11, CI [−0.19, −0.05]). The direct effect remained significant (*b* = −0.20, *p* = 0.03; total effect: *b* = −0.50, *p* = 0.002).

**Table 2.** Mediation model of the full sample (Study 2). *Note*. Delta method standard errors, bias-corrected percentile bootstrap confidence intervals, ML estimator.

	estimate	s.e.	z-value	*p*	95% confidence interval	
					lower	upper
direct effects						
Inequality_condition → WLB	−0.200	0.093	2.150	0.032	−0.379	−0.021
indirect effects						
Inequality_condition → StatusAnxiety_mean → WLB	−0.197	0.051	3.885	0.002	−0.310	−0.105
Inequality_condition → Competitiveness_mean → WLB	−0.107	0.034	3.093	0.003	−0.188	−0.049
total effects						
Inequality_condition → WLB	−0.503	0.105	4.787	0.002	−0.710	−0.219
total indirect effects						
Inequality_condition → WLB	−0.303	0.063	4.810	<0.001	−0.441	−0.189
residual covariances						
StatusAnxiety_mean 1 Competitiveness_mean	−0.375	0.055	6.824	<0.001	0.273	0.486

**Figure 6.**
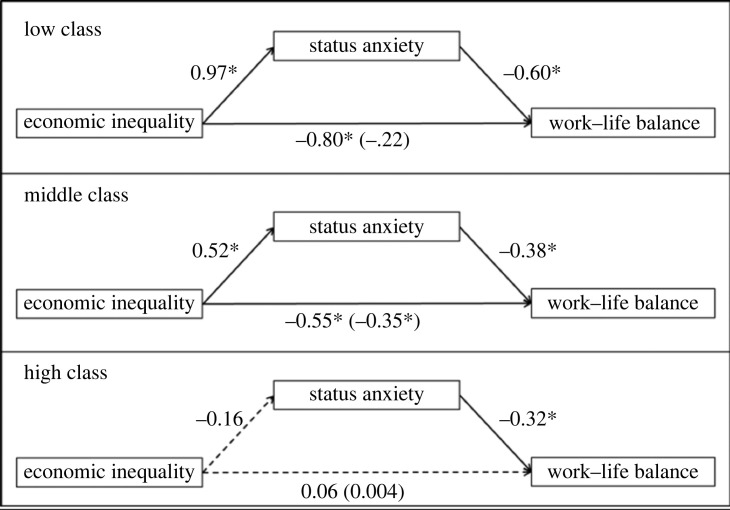
Path models for the assigned socio-economic classes in Study 2. Standardized coefficients are presented. Solid arrows and asterisks indicate significant paths. Asterisks indicate *p* < 0.05.

Finally, we tested the role of expected status anxiety separately as a mediator for all socio-economic classes (figure 6, tables 3–5). We did not include expected competitiveness in the model, as we did not find an interaction effect between inequality and socio-economic class for this variable. For the high-status group subsample (table 3), there is no evidence that the inequality manipulation has an effect on expected WLB (*b* = 0.06, *p* = 0.78). For the low-status group subsample (table 4), we found an indirect effect of the inequality manipulation on expected WLB through expected status anxiety (*b* = −0.58, CI = [−0.92, −0.34]), the partial effect was not statistically significant (*b* = −0.21, *p* = 0.20, total effect: *b* = −0.79, *p* = 0.002). Finally, for the middle-status group subsample (table 5), we found an indirect effect of the inequality manipulation on expected WLB through expected status anxiety (*b* = −0.20, CI = [−0.41, −0.06]), the partial effect remained statistically significant (*b* = −0.35, *p* = 0.04, total effect: *b* = −0.55, *p* = 0.006).

**Table 3.** Mediation model for participants assigned to the upper-class group condition (Study 2). *Note*. Delta method standard errors, bias-corrected percentile bootstrap confidence intervals, ML estimator.

	*β*	s.e.	z-value	*p*	95% confidence interval	
					lower	upper
direct effects						
high inequality → WLB	0.004	0.186	0.021	0.984	−0.374	0.405
indirect effects						
high inequality → status anxiety → WLB	0.052	0.065	0.804	0.421	−0.073	0.205
total effects						
high inequality → WLB	0.056	0.196	0.286	0.775	−0.344	0.474

**Table 4.** Mediation model for participants assigned to the low-class group condition (Study 2). *Note*. Delta method standard errors, bias-corrected percentile bootstrap confidence intervals, ML estimator.

	*β*	s.e.	z-value	*p*	95% confidence interval	
					lower	upper
direct effects						
high inequality → WLB	−0.214	0.167	−1.282	0.200	−0.565	0.118
indirect effects						
high inequality → status anxiety → WLB	−0.580	0.131	−4.431	0.002	−0.917	−0.343
total effects						
high inequality → WLB	−0.794	0.179	−4.438	0.002	−1.158	−0.448

**Table 5.** Mediation model for participants assigned to the middle-class group condition (Study 2). *Note*. Delta method standard errors, bias-corrected percentile bootstrap confidence intervals, ML estimator.

	*β*	s.e.	z-value	*p*	95% confidence interval	
					lower	upper
direct effects						
high inequality → WLB	−0.350	0.166	−2.110	0. 035	−0.696	−0.017
indirect effects						
high inequality → status anxiety → WLB	−0.198	0.079	−2.503	0.018	−0.406	−0.060
total effects						
high inequality → WLB	−0.548	0.173	−3.157	0.006	−0.893	−0.189

### 6.3. Discussion

Corroborating Studies 1a and 1b, Study 2 confirmed a lower WLB in the high-inequality condition. Moreover, we experimentally tested the role of socio-economic class, showing that participants assigned to the lower-class expected a greater imbalance between working and non-working activities when assigned to a high (versus low) unequal society.

We further found that enhanced status anxiety (in line with the pilot study) and competitiveness appeared as a class-specific mechanism and a general mechanism driving WLB, respectively. Specifically, high inequality enhanced the expected competitiveness for all classes, suggesting the perception of a broader competitive climate in this condition [82]. Conversely, status anxiety affected the lower class the most, followed by the middle class, with participants assigned to the wealthier group seemingly immune to inequality. Considering the lower classes' concerns, a coherent coping strategy would be to work longer hours and neglect other parts of life in an attempt to achieve more prestigious positions and not feel devaluated, in line with previous literature [50]. Furthermore, literature suggests that lower-income people are more sensitive to inequality, possibly making them more likely to suffer its negative effects [20]. Focusing on the effects of inequality for the wealthiest, upper-class participants were protected by their social standing, as their WLB did not decline in a highly unequal context.

## 7. Study 3

The aim of Study 3 was to replicate results concerning the principal path linking inequality to decreased work–life balance perceptions through increased status anxiety and competitiveness using pre-registered hypotheses and larger sample size. Since the results of Study 2 were satisfactory in giving us a picture of the role of social class in the relationship between inequality and work–life balance, we focused on the mediating path of status anxiety and competitiveness, adding working time (work hours and work days) as a second dependent variable, to see if it had a similar effect to the perceived WLB and be compared with the longitudinal data found in Study 1b.

Study 3 was pre-registered on the platform AsPredicted.com (https://aspredicted.org/7SG_HGY). As in the pilot study, we used the Bimboola paradigm [72] to manipulate inequality. We pre-registered eight main hypotheses, based on results found in the previous studies. H1: participants assigned to the high-inequality experimental condition would expect to work longer hours, compared with the people assigned to the low-inequality condition; H2: participants assigned to the high-inequality experimental condition would perceive less work–life balance, compared with people assigned to the low-inequality condition; H3: participants assigned to the high-inequality experimental condition would perceive increased status anxiety, compared with participants assigned to the low-inequality condition; H4: participants assigned to the high-inequality experimental condition would perceive increased competitiveness, compared with participants assigned to the low-inequality condition; H5: status anxiety would mediate the effect of inequality on working hours; H6: status anxiety would mediate the effect of inequality on perceived WLB; H7: competitiveness would mediate the effect of inequality on working hours; H8: competitiveness would mediate the effect of inequality on perceived WLB.

### 7.1. Method

#### 7.1.1. Participants

Five hundred Italian participants were collected using Prolific Academic through a Qualtrics link. Participation in the study was paid £1.50 and lasted 10 min. Following data cleaning (using the same exclusion criteria as in the pilot and in Study 2), we deleted data from one participant for failing one attention check and obtained a final sample size of 499 (248 men; 241 women; 10 non-binary, *M*_age_ = 31.34; s.d. = 10.03, age ranging from 20 to 64; more information about the sample is presented in table 1).

We determined the sample sizes by financial and methodological considerations. Our budget for this study allowed us to recruit 500 participants. This sample size allows us to detect a minimum effect of *d* = 0.26 for unidirectional *t*-test with alpha = 0.05, and beta = 0.90. In previous studies we conducted, we found that the effects of the inequality manipulation on work–life balance were *d* = 1.05 and *d* = 0.52. Similarly, in previous studies we found that the effects of the inequality manipulation on status anxiety were *d* = 1.69 and *d* = 0.47. Moreover, for the effect of the inequality manipulation on competitiveness we found *d* = 0.57. For what concerned the expected effect size for the indirect effect of inequality manipulation on WLB, in previous studies we found that the indirect effect of a mediation model with the inequality condition as predictor, work–life balance as outcome variable and status anxiety as mediator was *b* = 0.59 and *b* = 0.23. When these standardized betas are converted in Cohen's *d* using the ‘practical meta-analysis effect size calculator’ [83] the results are *d* = 1.49 and *d* = 0.48. Using the same procedure, we found that the converted Cohen's *d* for the indirect effect of the inequality manipulation using competitiveness as mediator was *d* = 0.41.

Thus, as *d* = 0.26 is around half of the smallest effect sizes found in the previous studies, we consider it sufficiently conservative as a minimum effect detectable.

#### 7.1.2. Materials and procedure

The procedure and the measures were the same as in Study 2, but we did not include manipulated socio-economic class, and thus a manipulation check for perceived wealth was not present.

Moreover, we also added a measure of working time to check whether results would be consistent with the ones found in Study 1b. To assess working time, we used two items developed ad hoc for this specific paradigm: ‘Imagining yourself as a citizen of Bimboola, how long do you imagine your workday to be (from one hour to 15 or more work hours)?’; ‘Imagining yourself as a citizen of Bimboola, how many days do you imagine working in a week (1 to 7 days)?’. The measures for perceived WLB (*α* = 0.93), status anxiety (*r* = 0.54), and competitiveness (*α* = 0.91) were the same as in Study 2.

### 7.2. Results

#### 7.2.1. Manipulation check

A Welch's *t*-test confirmed that participants perceived higher levels of economic inequality in the high-economic-inequality condition (*M* = 9.16, s.d. = 1.40), *t*_374.53_ = 27.098; *p* < 0.001; *d* = 2.43, compared with those in the low-inequality condition (*M* = 4.01, s.d. = 2.65).

#### 7.2.2. The effect of inequality on working hours, work–life balance perceptions, status anxiety and competitiveness

Through a correlational analysis we examined the association between variables related to working hours/days, the perception of work–life balance, and both inequality perception and status anxiety and competitiveness, reported in table 6. Results suggested that both working days/hours and perceptions of WLB were related to perceived inequality, status anxiety and competitiveness, although with stronger effects concerning perceived WLB.

**Table 6.** Correlational matrix Study 3 (Pearson's).

variables	1	2	3	4	5	6
1. inequality	—					
2. WLB	−0.336***	—				
3. working hours	0.317***	−0.408***	—			
4. days worked	0.293***	−0.323***	0.444***	—		
5. status anxiety	0.386***	−0.490***	0.313***	0.247***	—	
6. competitiveness	0.507***	−0.527***	0.341***	0.258***	0.671***	—

*p < 0.05; **p < 0.01; ***p < 0.001.

**Figure 7.**
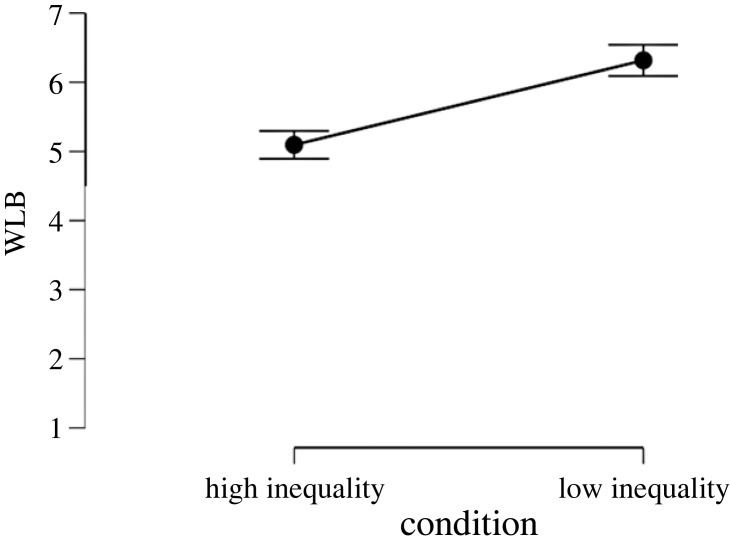
Effect of economic inequality on expected WLB in Study 3.

**Figure 8.**
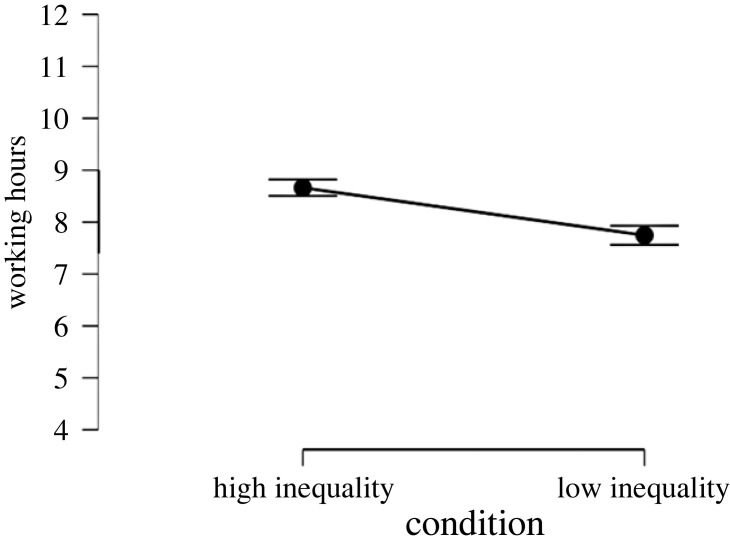
Effect of economic inequality on expected working hours in Study 3.

**Figure 9.**
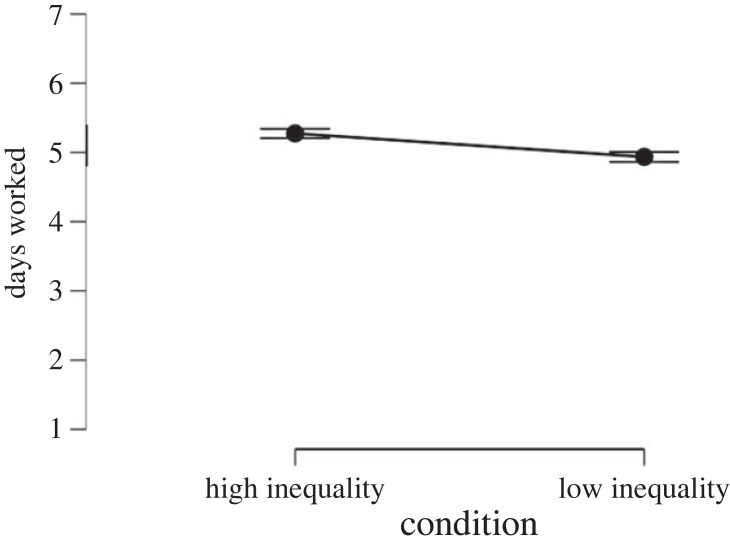
Effect of economic inequality on expected working days in Study 3.

To test the effect of inequality on working hours and WLB perceptions we ran two *t*-tests with the experimental condition as predictor and working time/WLB as outcome variables. Results showed that inequality has a strong effect on WLB perceptions (*d* = −0.96, *p* < 0.001, figure 7) hours worked per day (*d* = 0.67, *p* < 0.001, figure 8) and days worked per week (*d* = 0.61, *p* < 0.001, figure 9). That is, people assigned to the high-inequality condition expected to work longer hours (*M*_high inequality_ = 8.66, s.d._high inequality_ = 1.27, *M*_low inequality_ = 7.75, s.d._low inequality_ = 1.47), and more days (*M*_high inequality_ = 5.27, s.d._high inequality_ = 0.54, *M*_low inequality_ = 4.93, s.d._low inequality_ = 0.57) compared with people assigned to the low-inequality condition.

The effects of inequality manipulation on expected working hours (*d* = 0.66, *p* < 0.001) and expected working days (*d* = 0.60, *p* < 0.001) remained significant even after controlling for political orientation, subjective social class, type of job, educational level, gender and age.

**Figure 10.**
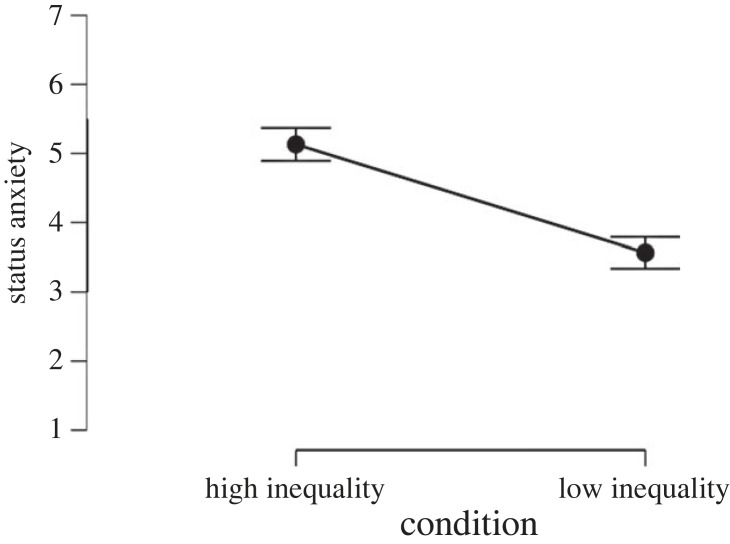
Effect of economic inequality on expected status anxiety in Study 3.

**Figure 11.**
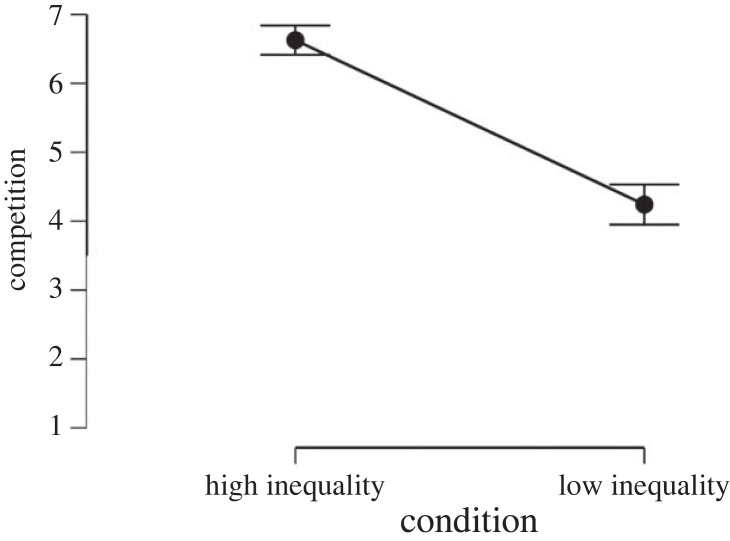
Effect of economic inequality on expected competitiveness in Study 3.

Moreover, people assigned to the high-inequality condition perceived less WLB (*M*_high inequality_ = 5.10, s.d._high inequality_ = 1.62, *M*_low inequality_ = 6.32, s.d._low inequality_ = 1.80) than people assigned to the low-inequality condition. Inequality also had an effect on both status anxiety (*d* = 0.83, *p* < 0.001, figure 10) and competitiveness (*d* = 1.17, *p* < 0.001, figure 11), with people assigned to the high-inequality condition perceiving enhanced status anxiety (*M*_high inequality_ = 5.13, s.d._high inequality_ = 1.93, *M*_low inequality_ = 3.57, s.d._low inequality_ = 1.82) and competitiveness (*M*_high inequality_ = 6.63, s.d._high inequality_ = 1.71, *M*_low inequality_ = 4.24, s.d._low inequality_ = 2.32) than people assigned to the low-inequality condition. The effects of inequality manipulation on expected WLB (*d* = 0.71, *p* < 0.001), expected status anxiety (*d* = 0.85, *p* < 0.001) and competitiveness (*d* = 1.16, *p* < 0.001) remained significant even after controlling for political orientation, subjective social class, type of job, educational level, gender and age. Finally, these results remained significant even after Bonferroni and Benjamini–Hochberg corrections were applied.

#### 7.2.3. Inequality increased working time and decreased work–life balance perceptions through status anxiety and competitiveness

**Figure 12.**
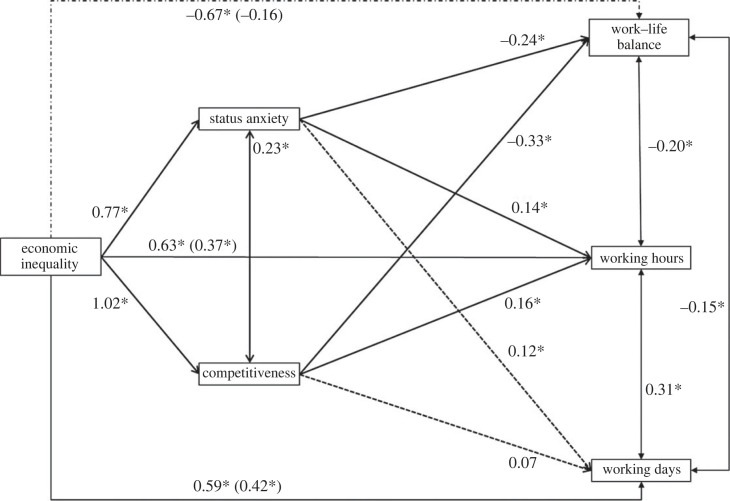
Mediation model examining indirect effects of economic inequality on expected WLB, working hours and working days, through status anxiety and competitiveness in Study 3. Standardized coefficients are presented. Solid arrows and asterisks indicate significant paths (*p* < 0.05).

We examined whether expected status anxiety and competitiveness mediated the relationship between inequality and WLB, working hours and working days using the software JASP [79]. We ran one mediation model with bootstrapping for 5000 resamples and 95% confidence intervals [80]. *p*-values were corrected using the Benjamini–Hochberg correction [81]. The model tested whether the inequality manipulation had indirect effects on the three output variables (WLB, expected working hours and expected working days), through different expected status anxiety and competitiveness together as mediator. We found support for indirect effects on WLB and working hours (all *b*s > 0.11, all *p*s < 0.05). However, for working days we did not find indirect effects of the inequality manipulation through expected status anxiety (*b* = 0.09, 95% CI = [0.00, 0.18]), nor through expected competitiveness (*b* = 0.07, 95% CI = [−0.04, 0.21]). Inclusion of political orientation, subjective social class, type of job, educational level, gender and age as control variables did not affect significance of tests results. The model is reported in table 7 and figure 12. Overall, results provide support for the pre-registered hypotheses.

**Table 7.** Mediation model Study 3 with expected status anxiety and expected competitiveness as mediators*. Note*. Delta method standard errors, bias-corrected percentile bootstrap confidence intervals, ML estimator.

	estimate	s.e.	*z*-value	*p*	95% confidence interval	
					lower	upper
direct effects						
high inequality → WLB	−0.157	0.086	−1.820	0.069	−0.333	0.014
high inequality → working hours	0.371	0.096	3.884	<0.001	0.165	0.569
high inequality → days worked	0.422	0.098	4.298	<0.001	0.228	0.608
indirect effects						
high inequality → status anxiety → WLB	−0.187	0.043	−4.294	<0.001	−0.299	−0.105
high inequality → competition → WLB	−0.329	0.060	−5.513	<0.001	−0.456	−0.211
high inequality → status anxiety → working hours	0.106	0.044	2.390	0.017	0.031	0.200
high inequality → competition → working hours	0.157	0.061	2.552	0.011	0.044	0.295
high inequality → status anxiety → days worked	0.090	0.045	2.004	0.005	0.001	0.178
high inequality → competition → days worked	0.073	0.062	1.180	0.238	−0.041	0.205
total effects						
high inequality → WLB	−0.672	0.084	−7.981	<0.001	−0.835	−0.507
high inequality → working hours	0.634	0.085	7.476	<0.001	0.464	0.803
high inequality → days worked	0.585	0.086	6.846	<0.001	0.418	0.761
total indirect effects						
high inequality → WLB	−0.516	0.060	−8.653	<0.001	−0.646	−0.403
high inequality → working hours	0.263	0.053	4.953	<0.001	0.162	0.382
high inequality → days worked	0.164	0.052	3.149	0.002	0.061	0.279
residual covariances						
status anxiety 1 competition	0.474	0.041	11.454	<0.001	0.402	0.556
WLB 1 working hours	−0.196	0.035	−5.596	<0.001	−0.277	−0.127
WLB 1 days worked	−0.156	0.036	−4.394	<0.001	−0.235	−0.084
working hours 1 days worked	0.315	0.041	7.638	<0.001	0.224	0.421
path coefficients						
status anxiety → WLB	−0.242	0.050	−4.834	<0.001	−0.358	−0.138
competition → WLB	−0.325	0.054	−6.075	<0.001	−0.435	−0.204
high inequality → WLB	−0.157	0.086	−1.820	0.069	−0.333	0.014
status anxiety → working hours	0.138	0.056	2.472	0.013	0.038	0.241
competition → working hours	0.155	0.060	2.601	0.009	0.043	0.279
high inequality → working hours	0.371	0.096	3.884	<0.001	0.165	0.569
status anxiety → days worked	0.117	0.057	2.051	0.040	0.015	0.221
competition → days worked	0.072	0.061	1.184	0.236	−0.041	0.199
high inequality → days worked	0.422	0.098	4.298	<0.001	0.228	0.608

### 7.3. Discussion

Results of Study 3 corroborated and replicated the results found in both correlational studies (Studies 1a and 1b) and experimental studies (pilot study and Study 2). In Study 3, participants assigned to the high inequality condition expected to work more hours/days (H1) and perceived lower WLB (H2). Moreover, we considered both status anxiety and competitiveness as potential mediators of the effect, in line with results found in the pilot study and Study 2. Specifically, our results showed an indirect effect of inequality on WLB and working hours and working days via status anxiety and competitiveness, in line with H5, H6, H7 and H8.

## 8. General discussion, limitations and practical implications

The ability to reconcile work with private life is fundamental for workers' well-being [1]. The focus of extant research on organizational features and job characteristics has hitherto given insufficient attention to key structural conditions shaping WLB, particularly economic inequality. With inequality rising across societies [16], greater empirical insights are needed concerning the possible interplay between inequality and individual psychological processes underpinning WLB, allowing for greater theoretical development and potentially more effective organizational interventions.

Two cross-country surveys established that people living in countries with higher inequality experience lower WLB (Study 1a) and higher number of hours worked (Study 1b). These results are consistent with the experimental evidence we gathered from a pilot study and two well-powered pre-registered studies (Studies 2 and 3) where the WLB of people living in a country described as highly unequal was inferred as lower than people living in a more equal context. Experimental data also revealed a possible explanation for this effect, namely increased status anxiety (all the three experimental studies) and competitiveness (Studies 2 and 3) triggered by inequality, in line with research in the field of social psychology [22,84]. Indeed, people in highly unequal situations were more likely to be concerned about their position on the social ladder and to perceive high competitiveness. This, in turn, affected their working time and their WLB perceptions. Study 2 further shows that the pattern concerning status anxiety is specific to low-class workers, while the effect of competitiveness applied for all classes. Individual difference of respondents, including their economic standing or their gender did not change the pattern of the results in the experimental studies.

### 8.1. Limitations and future directions

While results were consistent across the five studies, some limitations must be acknowledged. First, in experimental Study 2, respondents inferred that upper-class people have greater WLB. From a social psychology point of view, it is important to note that inferred WLB in the experimental scenarios may be due to the general tendency of people to reason through stereotypes while role-playing in the assigned scenario. In this context, when asked to identify with an upper-class or lower-class citizen of a fictitious society, participants possibly overestimated the positive features of a wealthy life, instead worsening expectations related with lower-class individuals. In fact, in contexts of high inequality, class stereotypes become more salient [85]. Future studies are needed to explore the link between respondents’ socio-economic standing and WLB across countries with different levels of economic inequality.

The role of individual factors on our experimental studies was also particularly intersting, as none of them had an effect on our main variables, although past research has highlighted the centrality of certain individual characteristics in shaping people's WLB, such as gender and socio-economic status. Concerning gender, for example, it has been found to be a key element for WLB (e.g. [86]), with recent empirical work on the value workers place on having work commitments ‘fit’ with family and personal commitments outside of work, suggesting women place more value on fit than men [33]. These gender differences were found to be similar across countries. The fact that such individual differences did not emerge in this controlled context is potentially because the manipulation was cancelling out gender differences in perceived economic inequality, since it explicitly indicated the level of inequality with concrete examples. The iterature suggests that women and men pay different attention to wealth cues, and this possibly affects their inferences regarding wealth differences. In the experimental paradigm, wealth cues were explicitly provided and were equal for every participant. Moreover, the results showed that once inequality is made evident, no gender differences emerge in the appraisal of its consequences. Further studies should address this specific claim, to better capture the complexity of this relationship.

Another important limit that could be addressed by future research concerns the lack of knowledge we have in relation to perceived social mobility in the manipulated scenario. In fact, working hours and perceived WLB could vary depending on the expectation of social mobility—or immobility—associated with high or low inequality of a certain context [87].

At the same time, expectations toward institutions could also affect the perception of individual WLB. Specifically, research shows that WLB varies by country and welfare state regimes, with better welfare states promoting better WLB [88]. Future research may look at the potential moderating effect of welfare state on the relationship between economic inequality and WLB.

Another limitation concerns that the experimental work was only conducted in one country, leaving room for cross-cultural differences in how inequality translates into WLB. For example, it is possible that the relationship between inequality and WLB relies more on competitiveness in individualistic societies than in collectivistic ones [89]. Moreover, caution is needed when interpreting the role of status anxiety and competitiveness in mediating the relationship between inequality and WLB/working hours. Indeed, the literature suggests that statistical models cannot provide evidence for causality *per se* where the mediator and outcome are measured simultaneously (as in Studies 2 and 3, see [90]).

It is also important to consider that status differences within an organization could influence the analysis of WLB outcomes. Future research can be improved by working to disentangle occupational status within organizations from general socio-economic status.

Concerning the variation in how work–life balance was measured across the four studies, findings indicated that perceived work–life balance is negatively correlated with time devoted to work. The correlation is small enough to conclude that the two indicators tap into related yet different facets of WLB. Interestingly, inequality had a more pronounced impact on individuals' perceptions of work–life balance, while its influence was less pronounced when examining more objective indicators such as working hours and workdays. The particularly notable difference concerns the days worked per week. This difference potentially stems from the small variability in the number of days worked per week, as compared with more dynamic indexes, namely hours worked per day and subjective WLB. The big effect concerning subjective WLB is also coherent with the literature conceptualizing the notion of well-being as core to the experience of being well people have, namely inherently subjective [91]. In this perspective, behavioural indexes have the crucial strength of being objective, yet they provide an indirect measure of the construct but do not encompass the construct itself. While results remained consistent whichever operationalization was used, greater cross-disciplinary research is needed to reach a broader consensus on the conceptualization and measurement of this term (see [92] for an overview in psychology) as well as longitudinal data efforts to provide consistent measures across time, particularly given its increased importance in policy and organizational debates.

### 8.2. Theoretical and practical implications

Our findings have significant theoretical and practical implications. Theoretically, these results suggest greater attention is needed for conceptualizing the complexity of WLB in relation to structural factors, like economic inequality. Exploring the effect of national contexts in shaping working outcomes is fundamental because organizations are central to countries' economies. Organizations create and distribute wealth [93], and as such, can exert power to reduce or perpetuate economic inequality. At the same time, as suggested by our findings and previous literature (e.g. [17]), organizational behaviour can be affected by the economic structure present in a country as well. Using the findings presented here, the integration of structural factors such as economic inequality (e.g. Bronfenbrenner's social-ecological model, [9]) can enrich future theoretical development in the field of organizational psychology, particularly by accounting for differences in perceived versus actual inequality, and the varying role of socio-economic class.

From a practical standpoint, governments and private organizations, as well as other stakeholders interested in promoting and implementing policies to improve WLB, can tailor the development of policies to address the role economic inequality and socio-economic class play in shaping working habits and expectations. For example, organizations could provide more tools to help workers reconcile work and private life in high-inequality contexts or promote more autonomous working contexts, given the relevance of autonomy for WLB [32]. They could also implement interventions aimed at reducing status anxiety and competitiveness, both of which result from economic inequality [22] are precursors of longer working hours [50], and are key variables in the relationship between inequality and WLB (Study 3). In addition, organizations in highly unequal countries could pay more attention to low socio-economic status workers, especially because low-status workers are often imagined in a dehumanized way and are inclined to accept their disadvantaged status quo [94], making it difficult for them to identify an unhealthy work situation.

Given growing economic inequality in industrialized societies, the empirical, theoretical and practical insights provided here may help facilitate government and organizational efforts aimed at improving workers' WLB.

## Data Availability

Data files and materials associated with the manuscript are openly available online on OSF (https://osf.io/3a6w9/?view_only=eb5e75fb0cff4314b31574588aca7f9f). The data are provided in electronic supplementary material [95].

## Footnotes

1 Since the measure included both expected normative competitiveness among Bimboola citizens and participants' own expected tendency to compete with other Bimboola citizens, we ran explorative factor analyses for Study 2 and Study 3, where parallel analysis was used as the extraction method, and Promax was used as the rotation method. We found a one-factor solution that explained 56% (in Study 2) and 70% (in Study 3) of the variance.

2 The factorial structure was coherent with the one of the original study, in all the experiments.
